# Research on value co-creation mechanism of platform enterprises in digital innovation ecosystem: A case study on Haier HOPE platform in China

**DOI:** 10.3389/fpsyg.2022.1055932

**Published:** 2022-11-25

**Authors:** Yuhua Li, Kaiwen Fu, Xiheng Gong, Ziwei Xiang, Jingyi Zhang, Chengjun Liao

**Affiliations:** School of Economics and Management, Shanghai Institute of Technology, Shanghai, China

**Keywords:** value co-creation, digital innovation ecosystem, platform enterprises, event system theory, digital innovation

## Abstract

Digital technology has given the innovation subject a new way of value creation, expanded the existing innovation ecosystem theory, and triggered scholars’ in-depth thinking on the digital innovation ecosystem. Based on the event system theory and taking Haier’s hope platform as a vertical case study, this paper deeply explores the research mechanism of value creation of platform enterprises in the digital innovation ecosystem, and reveals the role and impact of digital innovation ability, openness, and business innovation model on the process of co-creation. The research results show that: in the open connection stage, the platform solves the problem of weakening the advantages of the platform, and improves the innovation efficiency of enterprises by continuously improving the digital innovation ability; in the interactive and iterative stage, the platform carries out open innovation, breaks through the difficulties of platform expansion, and realizes the benign expansion of the platform. In the co-creation stage, the user experience is blocked, and the platform adopts the platform community business model to connect the user relationship and improve the user experience. In the digital innovation ecosystem, platform enterprises gradually form self-organization and self-circulation value co-creation through internal self-construction and external cooperation, and form a data-driven co-creation model.

## Introduction

With the rapid development of the digital economy, the fourth industrial revolution with digital technology as the core driver is reconfiguring and expanding traditional industries. At the same time, digital technology has created a powerful engine for expanding and improving traditional innovation development theories (Zhang et al., 2021). Digital technologies have given innovation agents new ways of value creation, expanded existing innovation ecosystem theories, and triggered scholars to think deeply about digital innovation ecosystems. Traditionally, an innovation ecosystem is defined as a system composed of heterogeneous subjects and their environment that work in concert to achieve innovation and value creation (Chen et al., 2022). With the development of the new technological revolution, the emergence of digital technologies has enriched the complex connection between innovation agents and innovation ecosystems. It also accelerates the digitalization process and the deep integration between innovation agents. In this context, the digital innovation ecosystem is defined as a complex economic structure in which organizations and individuals interact with each other. They rely on digital technologies to promote collaborative innovation in products and services (Zou et al., 2022). The digital innovation ecosystem not only introduces data as a factor of production, but also enhances the connections between subjects, promotes synergy among elements, and finally causes changes in the system logic.

A platform enterprise is a trading space for buyers and sellers to form an interconnected ecosystem (Du et al., 2022). It is based on platform users, creating value for them, delivering value, and capturing value. In the era of the digital economy, platform enterprises are rapidly emerging as the main support of the digital innovation ecosystem. The platform architecture they provide constitutes the foundation of the ecosystem. In the digital innovation ecosystem, it not only integrates resources efficiently for complementary enterprises by its unique dominant position, but also realizes value creation in the process of multi-agent interaction. In the face of strong external impacts such as the COVID-19 epidemic, the company can show the organizational toughness and anti-vulnerability ability that traditional enterprises cannot match, and show strong value creation ability and digital competitiveness. In the post-epidemic era, DingTalk focuses on building a benign platform-based ecosystem for efficient resource allocation. It used the cognition and experience brought about by trial-and-error learning to achieve effective interaction of the platform ecosystem behind hardware and software. And it assisted people to telecommute from home, while enterprises also promote teacher and student interaction by building virtual classrooms, contributing to epidemic prevention and control and corporate return to work. General Secretary Xi Jinping pointed out in the 20th National Congress that “building a modern industrial system and accelerating the construction of digital China.” With the tide of the digital economy, enterprises have the opportunity and ability to set up platforms to share information resources. And it builds digital innovation ecosystems with the help of platforms to achieve value creation. For example, Apple’s iPhone ecosystem can not only clarify customers’ propositions, but also stimulate customers’ needs by relying on products and services. Ding Talk, Apple, and other enterprises provide value returns for other organization members in the ecosystem by establishing their network platforms and improving their platform technologies. Based on the integration of rich complementary resources, platform enterprises provide users with rich and diverse function choices to establish an ecological cycle within the enterprise system. However, even if platform companies build a digital innovation ecosystem centered on themselves, the entire digital ecosystem is still at risk of failure. If they fail to handle the integration of resources between platforms and the mprovement of ecosystem governance, the whole digital ecosystem will still face he risk of failure. For example, traditional giants Sony and BlackBerry were dismally defeated in the competition with platform organizations such as Apple. Because they failed to effectively coordinate their relationships with other organizations in the system. How to deal with the relationship between platform enterprises and platform participants to promote resource sharing among ecological groups? How to realize co-creation among enterprises? Those phenomena have become the practical problem that platform enterprises are currently facing.

Digital platform enterprises spawned by digital technology not only promote the interaction and collaboration of system members, but also promote the deepening application of the digital innovation ecosystem, and it finally realizes the common evolution among innovation subjects (Bu and Chen, 2020). A large number of studies have been conducted in the literature on the evolution of competitive and cooperative relationships between platform enterprises and complementary players in the system (Peng and Yao, 2022). They also carried out a lot of research on the collaborative empowerment of platform-based enterprises to explore the connection between platform enterprises and the innovation ecosystem (Feng et al., 2022). And their fruitful research results provide a solid foundation for this paper to study the value co-creation mechanism of platform enterprises in the digital innovation ecosystem. However, under the rapid development of the digital economy, the following deficiencies still exist in this research area: how do platform enterprises play their roles in the complex network of platform relationships? How to use digital technology to integrate the resources between platforms and enterprises for value co-creation? These practical questions are in urgent need of theoretical responses.

To sum up, this paper combines the event system theory and case study methods. It takes the platform enterprises in the digital innovation ecosystem as the research object to explore resource interaction and explore the co-creation mechanism between platform enterprises and enterprises that can provide a solid and rich theoretical basis for relevant research on the digital innovation ecosystem. At the same time, this paper helps platform enterprises to further expand their main advantages and promote the sustainable and steady development of the innovation ecosystem. On the whole, it has important practical guiding significance for enterprise units to realize co-creation in the digital innovation ecosystem.

## Theoretical foundation and literature review

### Event system theory

Event System Theory (ETS) is a management theory proposed by Morgeson et al. (2015), which is applied to explore the degree of influence of temporal, spatial, and intensity attributes of events on relevant subjects. Currently, scholars commonly apply event system theory to qualitative research, which focuses on three levels: individual, organizational, and environmental. At the individual level, Zhao and Ren (2018) used event systems theory to explore in depth the process mechanism of entrepreneurial competence formation in a complex environment, and they analyzed the interactive effects of passive and active events on entrepreneurs’ ability to shape innovation. At the organizational level, Zhang et al. (2020) selected two local manufacturing enterprises, Gree and Geely, for a longitudinal case study to summarize the development pulse of corporate innovation quality and reveal the role of innovation atmosphere, innovation capability, and innovation openness in innovation quality improvement. At the environmental level, Li (2021) used a combination of “Solow’s residual value method” and event system theory to systematically interpret the causes of total factor productivity changes over the same period and summarize the trends and development requirements of technological progress.

### Literature review

#### Co-creation

**Figure 1:**
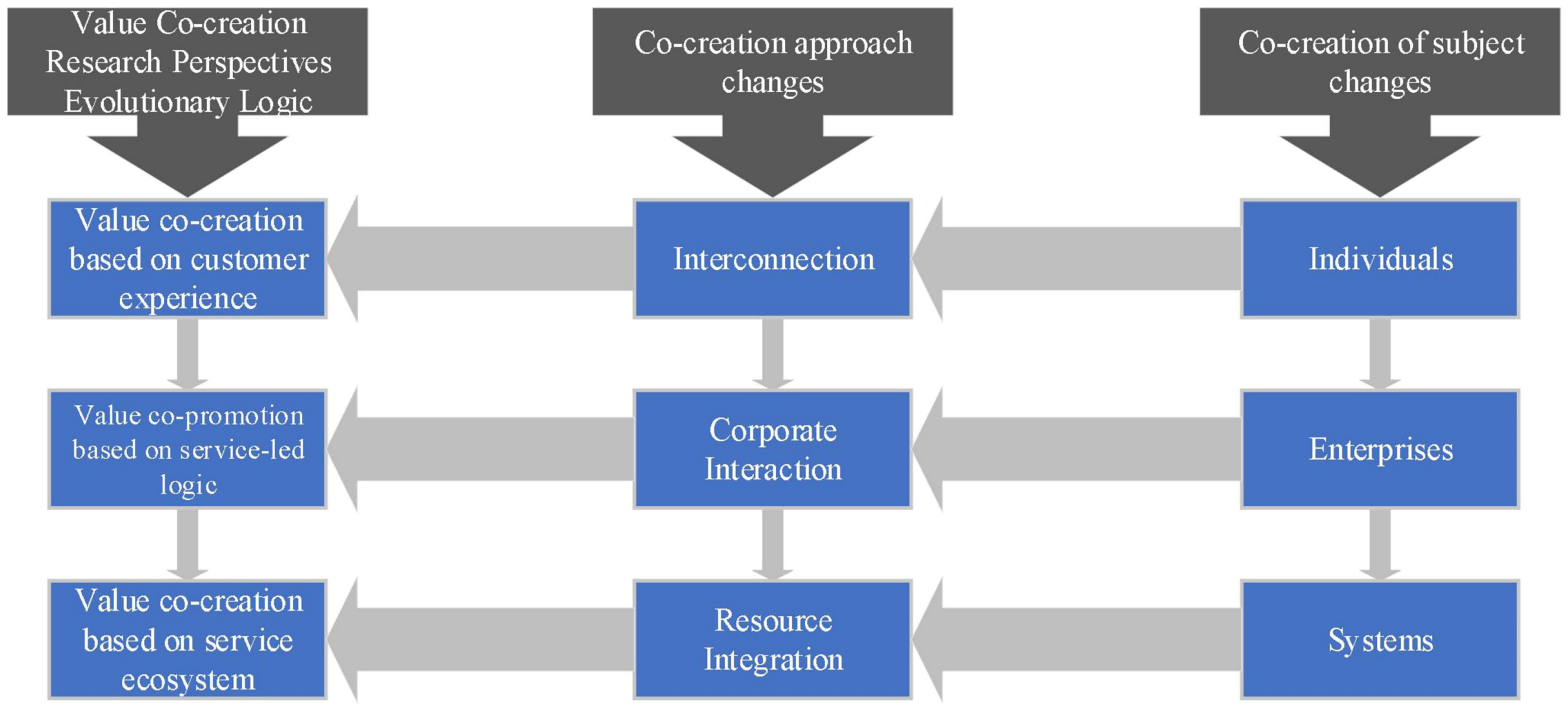
Evolutionary logic of value co-creation research perspective.

Value co-creation is the process of acquiring mutual value by creating subjects through exchanging services and integrating resources (Zhong et al., 2020). At present, the overall research framework of scholars on value co-creation focuses on three aspects. One is value co-creation based on customer experience. Zhou et al. (2022) choose the perspective of deep experience and interaction to explore the positive impact of customer psychological ownership on value co-creation, and they argue that companies can enhance value co-creation through customers’ sense of belonging and tacit understanding (Figure 1). The second is value co-creation based on service-led logic. The service-led logic emphasizes that value co-creation is achieved by the evolution of resource integration and capability synergy between enterprises and stakeholders. They also argue the focus of the resource integration and capability synergy process varies among different types of enterprises (Yao et al., 2022). Third, value co-creation is based on the service ecosystem. The number of co-creators within the service ecosystem is increasing. The co-creation of its participants is a dynamic evolutionary change process, and the co-creation relationship is developing toward a diversified trend, which itself focuses on data collection and utilization, and it can be more resilient and self-regulating on a larger scale (Wei and Liu, 2022).

Given the importance of value co-creation, there is also a large body of research centered on the results of the impact of value co-creation on co-creators. Firstly, at the individual level. The convenience and security of the platform factors positively influence not only customer satisfaction but also the intensity of continued use, and customer satisfaction is mediated between the platform factors and the intensity of continued use by customers (Zhu et al., 2022). Secondly, the organizational level. Wu and Tsai (2022) point out that platform companies need to understand producers and consumers with data tracking and in-depth analysis to provide them with innovative services. In this way, user satisfaction, loyalty, and frequency of use are improved, and a virtuous circle of value co-creation is eventually established. Finally, at the system level, value co-creation through user participation is based on the logic of community interaction, which integrates producers and demanders. Community management becomes an important source of value acquisition and drives the digital platform ecosystem to realize value co-creation through cross-border integration (Zhang et al., 2022).

#### Digital innovation ecosystem

**Figure 2:**
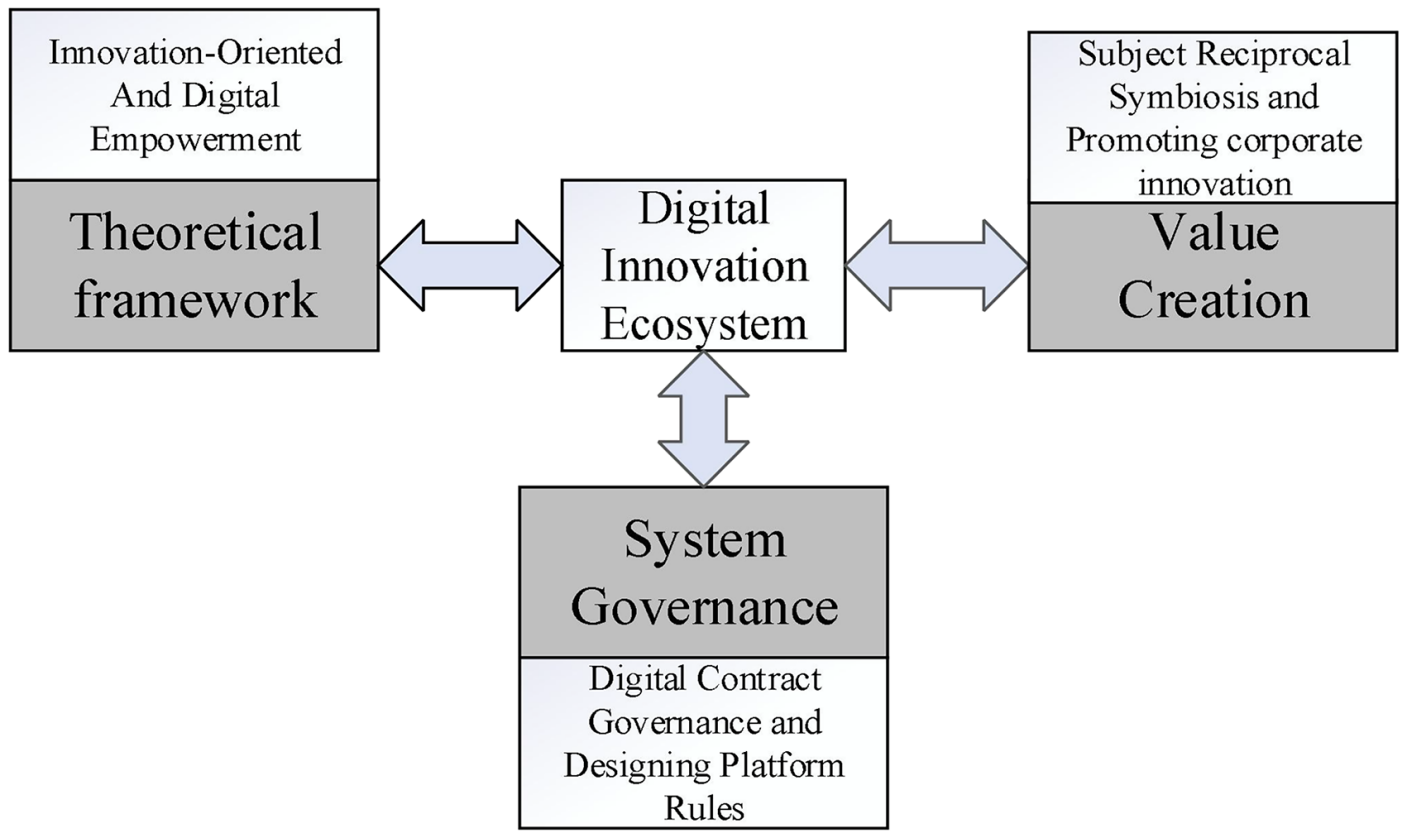
Digital innovation ecosystem research analysis chart.

In the digital era, data-driven innovation has a different innovation logic from technological product innovation in the industrial economy form and presents the ecological structure and operational characteristics. Research on the digital innovation ecosystem focuses on three aspects: system development, value creation, and organizational governance. First, exploring the theoretical framework of the digital innovation ecosystem. Zhang et al. (2021) believes that the digital innovation ecosystem should be divided into two categories: innovation-oriented and digital empowerment. The innovation-oriented digital innovation ecosystem is mainly centered on digital subjects and aims to facilitate digital innovation generation, diffusion, and application. On the other hand, the digital innovation ecosystem of digital empowerment is the result of the deep integration of the digital process and value co-creation among innovation subjects. It aims at the all-around digital transformation of the subject, structure, system, and function of the innovation ecosystem. Secondly. Value creation emphasizes that innovative subjects form a multilateral cooperative relationship based on a common vision. It is to build a digital innovation ecosystem of mutual symbiosis, virtuous circle, and win-win cooperation from the perspectives of multi-systems, multi-channels, and multi-subjects. In this way, it promotes the common evolution and value sharing of all parties (Ning et al., 2022). Building a digital innovation ecosystem can also help core enterprises to enhance their motivation to innovate, and promote the accumulation, integration, and absorption of innovation resources in the system. Finally enhance their core competitiveness (Zou et al., 2022). Third, the research related to the governance of the digital innovation ecosystem focuses on the characteristics and governance dilemmas of the system to make the activities more process-oriented and standardized for successful implementation. On the one hand, through digital contract forms such as smart contracts, the traditional contract governance means are expanded, which provides a new solution to the problem of intellectual property authorization and transfer in the system. And at the same time, it can reduce the opportunistic risk in innovative behavior (Pereira et al., 2019). On the other hand, the digital platform provides behavioral guidelines for a wide range of participating subjects by designing relevant operating rules. In this way, it improves the consistency and compatibility of the system’s innovative behavior and accelerates the speed of complementarity among participants (Figure 2). Ultimately, it promotes the establishment of one-to-many and many-to-many collaborative relationships (Wei and Zhao, 2021).

#### Platform enterprises

**Figure 3:**
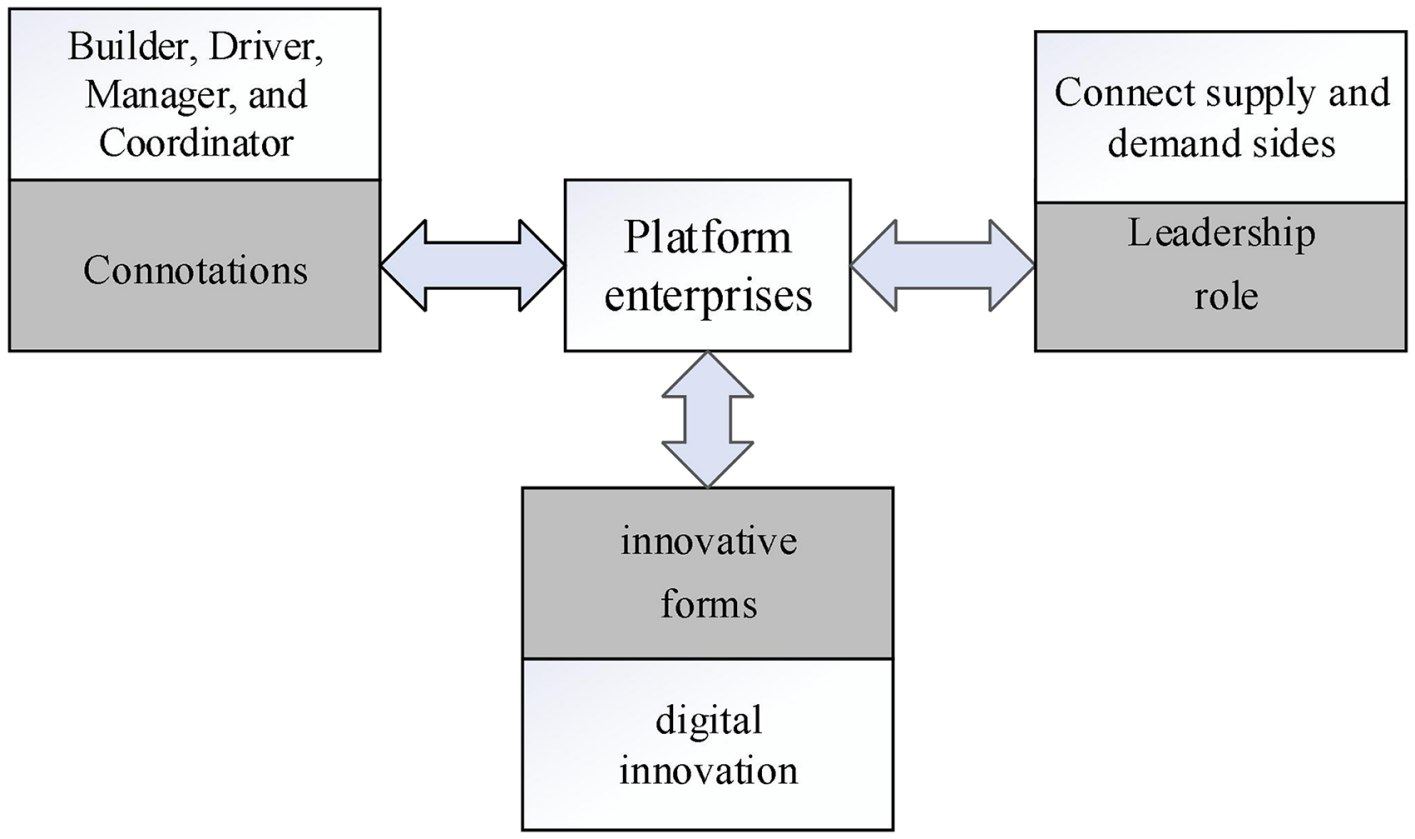
Research and analysis diagram of platform enterprises.

In the digital innovation ecosystem, platform enterprises are based on platform users and create value for them. Along with the increase of users, enterprises also generate network effects and positive feedback (Xing and Tang, 2021). As an important part of the innovation ecosystem, the current relevant research focuses on several aspects. Firstly, it is about the connotation of platform enterprises. The platform enterprise is not only the builder and driver of the system platform, but also the natural manager and coordinator of the ecosystem (Wang and Zhang, 2021), to realize the value creation in the interaction process of multiple subjects in the system. The second is about the leadership role of platform enterprises. Luo and Du (2018) dissects the inner operating mechanism of platform leadership embedded in substantive options, and he points out that platform enterprises use multilateral markets to connect supply and demand sides, ultimately improving the effectiveness of platform markets. Finally, it is about the innovative forms of platform companies. Beltagui and Rosli (2020) argue that the digital innovation ecosystem is a platform structure in that platform enterprises use digital innovation to provide the foundation for the ecosystem, and participate in the open innovation of the system, thus forming an ecological interactive circulation organization system (Figure 3).

In summary, many scholars at home and abroad have conducted a lot of research on co-creation, the digital innovation ecosystem, and platform enterprises from their perspectives, and their fruitful results are important for further exploring the co-creation mechanism of platform enterprises in the digital innovation ecosystem. However, through the analysis of the existing literature, the following deficiencies are still found in this research area: (1) the digital innovation ecosystem is a complex system composed of a large network of users and redundant enterprises, which needs to provide support for the interaction of participants with different motives. While the existing literature focuses on the influence factors of the creation of participants from a single perspective of participants, lacking the interaction between enterprises and enterprises. (2) The rapid development of new technology platforms has triggered scholars’ attention to the platform economy, and the existing literature mainly focuses on digital platforms and platform ecosystems, and platform economy. However, platform enterprises, as the main support of the digital innovation ecosystem, have fewer and more fragmented studies on resource interaction, resource integration, and value co-creation mechanisms between platform enterprises and enterprises.

Therefore, this paper adopts a combination of the single-case study method and event system theory to explore the co-creation mechanism of platform enterprises in the digital innovation ecosystem. It also enriches the research on the digital innovation ecosystem, helps platform enterprises expand their main advantages, and ensures the steady development of the innovation ecosystem.

## Research design

### Research methodology

This paper focuses on the mechanism of resource interaction and value co-creation between platform enterprises, which is an in-depth discussion on the development and application of the digital innovation ecosystem. When analyzing problems such as “HOW” and “WHY,” the single-case analysis method can better ensure the details of materials and research depth (Mao, 2020). A single-case study can have multiple analysis units, which can be regarded as a series of experiments to summarize a more reliable theoretical model, thus ensuring the credibility of the case study (Yang and Wei, 2021). The event system theory plays an important role in case and qualitative research, and it emphasizes that events are the external dynamic experiences of entities, including the interaction between entities (Morgeson et al., 2015). Therefore, this paper uses the theory of the single-case and event system to deeply discuss the realization mechanism of resource integration co-creation of platform enterprises in the digital innovation ecosystem.

### Case selection

The rapid development of platform economy provides rich and varied practical cases for this paper, such as Haier’s HOPE, platform ecosystem, Aerospace, Cloud Network INDICS, and so on. This paper chooses the digital innovation ecosystem of Haier Group (hereinafter referred to as Haier) as the research object, that is, Haier HOPE platform ecosystem with enterprises as the hub. HOPE Platform Ecosystem is an open innovation platform established by Haier R&D Center in October 2009. This platform adds innovation and digital technology to the relationship network between technology suppliers and demanders. It promotes the interaction between the two parties and the birth of innovative products. Since the HOPE platform was officially launched in 2013, Haier formally gathered the original external technical partners, manufacturers, global R&D centers, and small on the HOPE open innovation platform. Use digital technology to provide participants with all-around innovative services, integrate and innovate participants’ resources, and create value together. According to the development stages of coping with core problems and the evolution of operation mode in different stages. The life cycle of the HOPE platform is divided into five stages: Open Phase (2009.09–2010.07) to Break the closed R&D model; Interaction Phase (2010.07–2013.10) to build a global innovation resource network; Connection Phase (2013.10–2015.04) to collaborative innovation with external resources; Iteration Phase (2015.04–2018.07) to a positive cycle of open innovation system; and Co-creation Phase (2018.07-present) to co-creation and win-win sharing innovation ecology.

### Data acquisition

To improve the reliability and validity of the case study, this paper integrates various data sources for triangulation, including interview data collected by the data team and documents and materials directly obtained from the enterprises, which involve platform reform and digital innovation ecosystem construction, informal interview materials, corporate websites, media reports, and other secondary materials. After collecting the first-hand data, this paper divides the researchers into two groups. The two groups of researchers copy the collected audio and text content at the same time, and sort out and record them by text. Researchers analyze and discuss the sorted text content. This paper also obtains Haier’s second-hand information through other channels. Such as Haier’s official website about the HOPE platform system, related academic papers, dissertations, related books, critical articles, and media reports. Finally, the collected first-hand data and second-hand data are numbered, proofread, and sorted out, and the database of the digital innovation ecosystem is established, which provides convenience for this study.

### Key construction and description

This paper involves some key constructs such as event attributes (event strength, event time, and event space), digital innovation capability, platform openness (Gawer 2014a,b), platform business model innovation, etc. By combing the existing literature, the connotation of key constructs is clarified, and the following key constructs are described:

**(1) Event attributes.** The event theory can be divided into active events and passive events according to the different degrees of influence among related entities. With the help of time, space, and intensity, a three-dimensional system is constructed to explain the attributes of events. Referring to the research of Liu and Liu (2017), this paper holds that the event time includes time and duration; the intensity of events includes novelty, disruption, and criticality; and the space includes the origin, spreading range, and the distance between the entity and the event.

**(2) Digital innovation capability.** This paper examines the process of platform enterprises realizing the co-creation of enterprise units in digital innovation systems in dynamic events. Because digital innovation uses digital technology in the innovation process of exploration and utilization. It will give birth to a series of new ways of value creation and value distribution (Tilson et al., 2010). In this paper, the digital innovation ability is divided into the utilization of digital innovation ability and the exploratory digital innovation ability (Zhang et al., 2020).

**(3) Openness of the platform**. The different degrees of platform opening will affect the resource integration, innovation and development of enterprises, and the change of business model of enterprises. This paper holds that the development of an enterprise not only depends on the internal knowledge and resources of the organization, but also needs to obtain resources such as creativity and knowledge from the outside in an open environment to strengthen its capabilities and achieve better sustainable development.

**(4) Platform business model innovation.** An important way for the platform ecosystem to realize co-creation is platform business model innovation. It involves the benign interaction among various stakeholders in the ecosystem. It also fully mobilizes and stimulates resources and capabilities from all sides (Zhong et al., 2020), and truly embodies the concept of ‘co-creation’ in the implementation process. This paper holds that the innovation of a platform business model is the interaction and dynamic evolution of components and elements among platform enterprises with the evolution of time, and gradually matures.

### Event data coding analysis

This paper takes the key events during the development of the Haier HOPE platform as the basic unit and takes five stages (opening, connection, interaction, iteration, and co-creation) as the embedded analysis unit. According to the coding rules, the coding content of each event includes the event code (year, month, and day of the event), the classification and identification (event category), and the event description. This paper selects 24 events, which are divided into active events and passive events, and takes the occurrence of enterprise-led events as a measure. There are 24 event codes. The event code consists of two parts, the stage number plus the event occurrence time. For example, II20100105 indicates the event that occurred on October 5th, 2010 in the connection stage of the HOPE platform.

Referring to the research of Liu et al. (2017), this paper holds that event intensity includes three characteristics: novelty, disruption, and criticality. The spatial diffusion range is divided into: +++ reachable outside the industry; ++ reachable inside the industry; and + reachable inside the organization; event intensity is divided into three levels: +++ strong representativeness; ++ representative; and + Weak representation. The specific description and event attributes are shown in Table 1.

**Table 1:** Summary of key events in the growth of the HOPE platform.

Event codes	Event types		Event descriptions	Event intensity			Events space
	Passive	Active		Novelty	Subversive	Criticality	
I200910	√		Explore external technology and innovation resources around the world and establish Haier Open Innovation Center.	+++	+	+++	+
II201007		√	HOPE’s new portal was launched to publish requirements and match resources to form an innovative internal transformation method.	++	++	+	++
II20100105		√	The “Tail-less TV” was exhibited at CES; Haier Wireless, Haier’s first industrial micro, was successfully incubated	++	+	+	+
III201310		√	HOPE platform 1.0 was officially launched innovation exploration developed online and offline.	+++	++	+++	+++
III201310	√		The online version of the “Open Innovation Center” actually attracted fewer users than expected.	++	+	+	+
III201406		√	HOPE platform revamped and upgraded to HOPE 2.0	++	+	++	+
III201408		√	The platform to commercialize the technology.	+++	++	++	+++
IV20150423	√		HOPE platform is included as a Chinese case in the “China-China Technology Trade Report.”	+	+	++	++
IV20150507	√		HOPE platform welcomes its first third-party customer - Faurecia.	++	+	+++	++
IV20150807		√	Agent (Innovation Partner) program launched.	++	+	++	+
IV201608	√		The HOPE platform has become a benchmark case of innovation.	+	++	++	++
IV20161215		√	The “Innovation Partner Program” was upgraded to officially open the exploration of an innovative community model.	+++	++	++	+
V201807	√		HOPE platform signed a strategic cooperation agreement with IEEE.	+++	+++	++	+++
V20190621		√	HOPE held the “China-Japan Open Innovation Exchange.”	++	++	+	+++
V201911		√	HOPE platform officially opens the era of data-driven service upgrades.	++	+++	++	+
V202002		√	HOPE platform innovation activities officially entered the rapid promotion fission stage.	++	+	+	+
V202007		√	HOPE’s innovative service system, officially opened the exploration phase.	+++	++	++	++
V20210525	√		HOPE is integrated into the global innovation chain.	++	+	+++	+++
V20210709		√	HOPE enables users and resources to interact with each other at zero distance.	++	++	++	+

**Figure 4:**
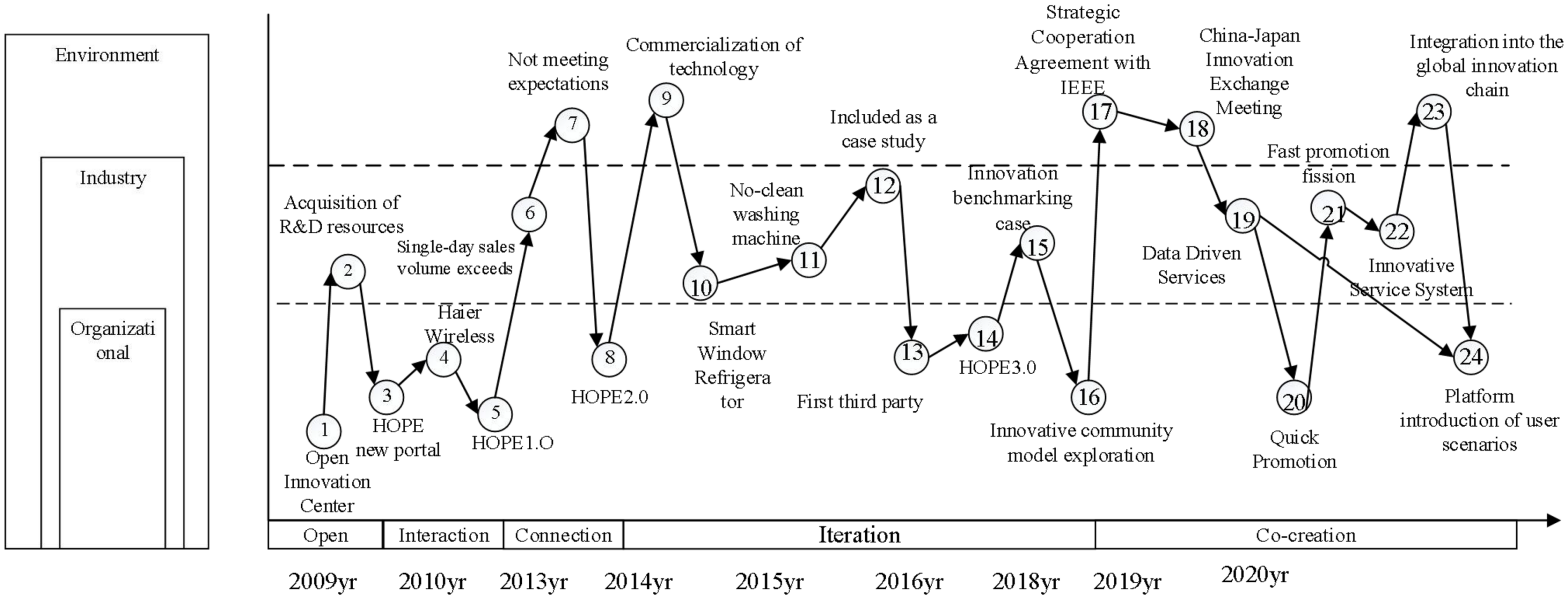
Key events of HOPE platform development.

The analysis level of this case study is the key events experienced in the growth of the HOPE platform. With the help of the occurrence of 24 key events, this paper attempts to analyze the internal mechanism of events’ influence on co-creation. Then, it also analyzes the influence relationship between digital innovation capability, platform openness, and business model innovation and finally summarizes the development context of key events, as shown in Figure 4.

## Case analysis and research findings

### Impact of digital innovation capabilities on co-creation of platform companies

Digital innovation capabilities can bring indirect innovation benefits to firms by reducing the cost and risk of their innovation activities. According to Harms (2015), platform market pioneers often lack innovation references and are less likely to act proactively, but only passively, like “confined to a single, coherent sphere of influence.” The emergence of reactive events has prompted platform companies to take action to improve their digital capabilities and to change their digital level to a certain extent. Therefore, they can better provide value to complementary companies and share value in the ecosystem they belong to. See Table 2 for a detailed analysis.

**Table 2:** Platforms use reactive events to enhance digital innovation.

Passive events	Evidence of individual cases					Follow-up initiative events	Digital innovation capability segmentation
	Research materials	Event intensity			Event space		
		Novelty	Subversive	Criticality			
I200910	Break the traditional closed model, explore external technologies and solutions around the world, and establish Haier Open Innovation Center.	+++	+	+++	+	I201007	Exploitability
III201310	The lack of maturity and innovation in digital technology has led to attracting fewer users than expected, and less than expected.	++	+	+	+	III201406	Exploitability
IV20150423	HOPE platform is included as a Chinese case in the “China-China Technology Trade Report.”	+	+	++	++	IV20150807	Exploitation and exploratory at the same time
IV20150507	HOPE platform welcomed its first third-party customer - Faurecia, and both parties signed a strategic cooperation agreement.	++	+	+++	++	IV20150807	Exploratory
IV201608	HOPE platform was selected as a benchmark case of innovation in the book “Innovation Management - Gaining Sustainable Competitive Advantage.”	+	++	++	++	IV20161215	Exploratory
V201807	HOPE platform signed a strategic cooperation agreement with IEEE.	+++	+++	++	+++	V20190621	Exploratory
V20210525	HOPE platform is driven by two wheels of science and technology innovation and results in the transformation to integrate into the global innovation industry chain.	++	+	+++	+++	V20210709	Exploratory

The negative reactive type of events put the HOPE platform under tremendous pressure at its creation, to apply digital technologies to update and transform its operating model, and to co-create value to other businesses through the transformed model. According to Kane et al. (2015), most platform companies are not prepared to deal with digital trends at the beginning of their establishment. In the absence of technology and resources, digital innovation capabilities are often developed in reactive-type events. And this phenomenon is reflected in many events of the company, for example, like Event I200910. In the new economic form, the problems of uncertainty and lag in R&D and lack of digital innovation capability also exist in Haier. However, after major strategies such as networking, Haier began to emphasize open innovation and the use of digital technology. And exploring external technologies and solutions worldwide and establishing the Haier Open Innovation Center. In 2013, Haier’s HOPE platform was officially launched, but initially, it was more like an online version of the “Open Innovation Center.” And the lack of digital technology and innovation capabilities led to a decrease in the number of users participating in the platform. Therefore, the company is in the negative impact of reactive events, through the use of digital innovation capabilities to build a new mode of operation. Therefore, the platform can improve its capabilities to ensure the transfer and sharing of resources within the system, and with further integration to optimize the allocation, it can be better for other companies in the system to create value and share value. As a result, this paper proposes the following propositions.

*Proposition 1a*: In the open and connected interactive phase of the enterprise, reactive events drive companies to improve interfirm co-creation with the help of exploitative digital innovation capabilities.

It has been shown that the scope, functionality, and value of digital offerings continue to evolve even after the innovation is launched or implemented (Lakhani et al., 2013). Most platforms are still dysfunctional in terms of service content when they are first introduced and are in a constant state of change. The scope and scale of innovation will expand with the various entities involved in innovation. This case study finds that reactive event intensity and spatial diffusion will have an impact on the digital innovation capabilities of firms to some extent. In the iterative phase, firms tend to use their exploratory digital innovation capabilities to cope with passive-type events with high intensity and wide spatial diffusion, to find innovation opportunities that integrate resources, and to improve the efficiency of resource sharing, to better realize co-creation. This feature is reflected in the events IV20150423, IV20150507, and IV201608 in this case. For example, in event IV20150507, the Haier Open Innovation HOPE platform and Faurecia reached an agreement, and the platform welcomed its first cross-border third-party customer. On the same day, the two parties signed a strategic cooperation agreement on the integration and sharing of regional innovation resources. They said that in the future, they will achieve advantages and resource sharing in the automotive field through cross-border cooperation. The Haier HOPE platform integrates the world’s best resources and provides a stage for technological innovation to generate ideas. It also quickly matches these ideas with related companies and turns them into innovative products for the first time, to accelerate the innovation and upgrading of enterprises. Ultimately, it provides wider resource channels for other enterprise users in the system, to realize resource integration more quickly. As a result, this paper proposes the following propositions.Proposition 1b:Passive-type events change the innovation capability of enterprises and influence the transformation of enterprises from exploitative digital innovation to exploratory digital innovation capability.

### Impact of the degree of platform openness on the co-creation of platform enterprises

To meet the better and sustainable development of enterprises, platforms not only rely on continuous collaboration to acquire key digital resources (compatibility), but also share open platform interfaces and standards with partners. Finally, the platform is expanded by driving a shift in the platform’s digital innovation model (Tilson et al., 2010). When enterprises are initially established, they usually rely on the knowledge and resources within the organization to explore and seek opportunities. Companies become progressively closed in the long run, with slow development and poor prospects for high-risk exploratory strategic activities. For example, in events I200910 and II201007, the HOPE platform initially had a low degree of openness. And it was faced with the problems of lack of resources and backward production at the same time. Haier used the ability to leverage innovation to deal with reactive events. It decided to establish an innovation center and emphasized open innovation. Meanwhile, it grasped user needs and integrated global resources to realize “the world is my R&D department, The world is my R&D department” finally.

When the degree of openness of the platform is increasing, the company continuously obtains resources from outside and then integrates them with internal resources to achieve better and sustainable development. When the degree of openness of the platform is relatively high, it means that the enterprise has more high-quality resources and opportunities. At this time, the enterprise usually adopts the exploratory innovation ability to deal with passive events. Such as event IV201608. HOPE platform was selected as an innovation benchmark case in the book “Innovation Management - Winning Sustainable Competitive Advantage.” With a high degree of openness, the platform used its resources to upgrade the “Innovation Partner Program” when they face the impact of competitors and formally opened the innovation community model exploration. In this upgrade process, the participants not only expanded from institution to individual, but also evolved from simple demand release to community interaction and resource matching. This process makes it an in-depth exploration of innovative community models. As Mr. Zhang, Chairman of the Board of Directors of Haier Group, said, “There is no successful enterprise, only the enterprise of the times, and the so-called success is only to step on the beat of the times.” Enterprises should constantly subvert themselves, transcend themselves, and make open innovation for all new products and services. The above phenomenon is also reflected in the response to reactive events such as event V20210525, as shown in Table 3. As a result, this paper proposes the following proposition.Proposition 2:In the case of low openness, firms tend to adopt exploitative innovation capabilities in response to reactive events; in the case of high openness, firms tend to adopt exploratory innovation capabilities in response to reactive events.

**Table 3:** Impact of events on the degree of openness of firms.

Events	Research materials	Degree of openness	Follow-up events	Subsequent events role
III201310	The platform released the online version of “Open Innovation Center,” which attracted fewer users than expected	Low	III201406	HOPE was revamped and upgraded, adding a news module and innovation community to better realize zero-distance interaction between users and resources on the platform.
V20210525	After the cooperation between HOPE and Silicon Valley High Innovation Council, the platform is driven by two wheels of technology innovation and achievement transformation to integrate into the global innovation industry chain.	High	V20210709	HOPE is centered on creating a user experience, taking the scene applications as the traction, breaking industry boundaries, and carrying out more cross-border integration and innovation.

### The influence of platform business model innovation on co-creation of platform enterprises

The innovation of the platform business model is different from the general traditional business model. The traditional business model emphasizes the needs of users and their own development needs, while the platform business model emphasizes collaborative innovation and value co-creation. At the beginning of HOPE, the first task of the platform is to accumulate resources and gain viability. Such as events I200910 and II201007. During these periods, enterprises focused on manufacturing to accumulate resources to meet their own development needs. Business models are also relatively backward and lack innovation. And with the continuous development of enterprises and the emergence of innovative resources. The platform began to implement the value proposition of development-oriented win-win and enterprise empowerment. Through the exploration and development of open innovation, the platform not only empowers the production technology of enterprises, but also realizes the wisdom, knowledge, and skills. Such as event III201406, the platform not only broadens the traditional business model, optimizes, and upgrades the platform, but also adds a new module and an innovative community. And finally, the platform achieves zero-distance interaction between users and resources. Platform companies and communities inspire common ideals and grow together in a win-win situation. At the same time, the platform stimulates the vitality of community enterprise organizations and forms an empowering value proposition. Therefore, this paper proposes the following propositions:Proposition 3a:The value proposition of win-win and empowerment drives the transformation of enterprises, prompting them to shift from the open stage to the interactive stage.

Diversified information technology breaks down the barriers of time and space between individuals and expands the scope of traditional communities. And it enables people across geographical boundaries to communicate and share information and knowledge with each other. In the event IV20161215, the HOPE platform is proposed to officially open the exploration of the innovative community model. The innovation community uses the “micro-insight” tool to conduct precise user behavior insights to define the real needs of users. In this way, we can provide user data support for customers to carry out product innovation and explore innovation opportunities and innovation directions. The platform community brings the relationship between the platform and users closer, and platform companies optimize service quality around user needs, which improves user experience. Digging deeper into the core, user needs can drive key user groups to join the platform, thus involving users at the other end through indirect network effects (Du et al., 2021). With clear service logic and meeting user needs, the user experience will be improved accordingly. On the one hand, through the community business model, the platform sorts out and refines user needs, and the business logic of platform services gradually becomes clear. On the other hand, through the relationship with users, common effective needs are identified and users’ needs are profoundly satisfied, which can also bring a better experience to users. Therefore, when considering co-creation, not only the users’ needs but also the platform’s business model should be taken into account. The platform optimizes and streamlines the business model around key user needs while retaining the core needs and coordinating the difficulties of different stakeholders. In this way, we can better promote user relationships, improve user experience, and build a relational platform ecosystem. Therefore, this paper proposes the following proposition.Proposition 3b:A high degree of platform-community business model innovation will promote ecological diversification, improve the experience of community partners, and accelerate the ecological operation.

### Research findings

**Figure 5:**
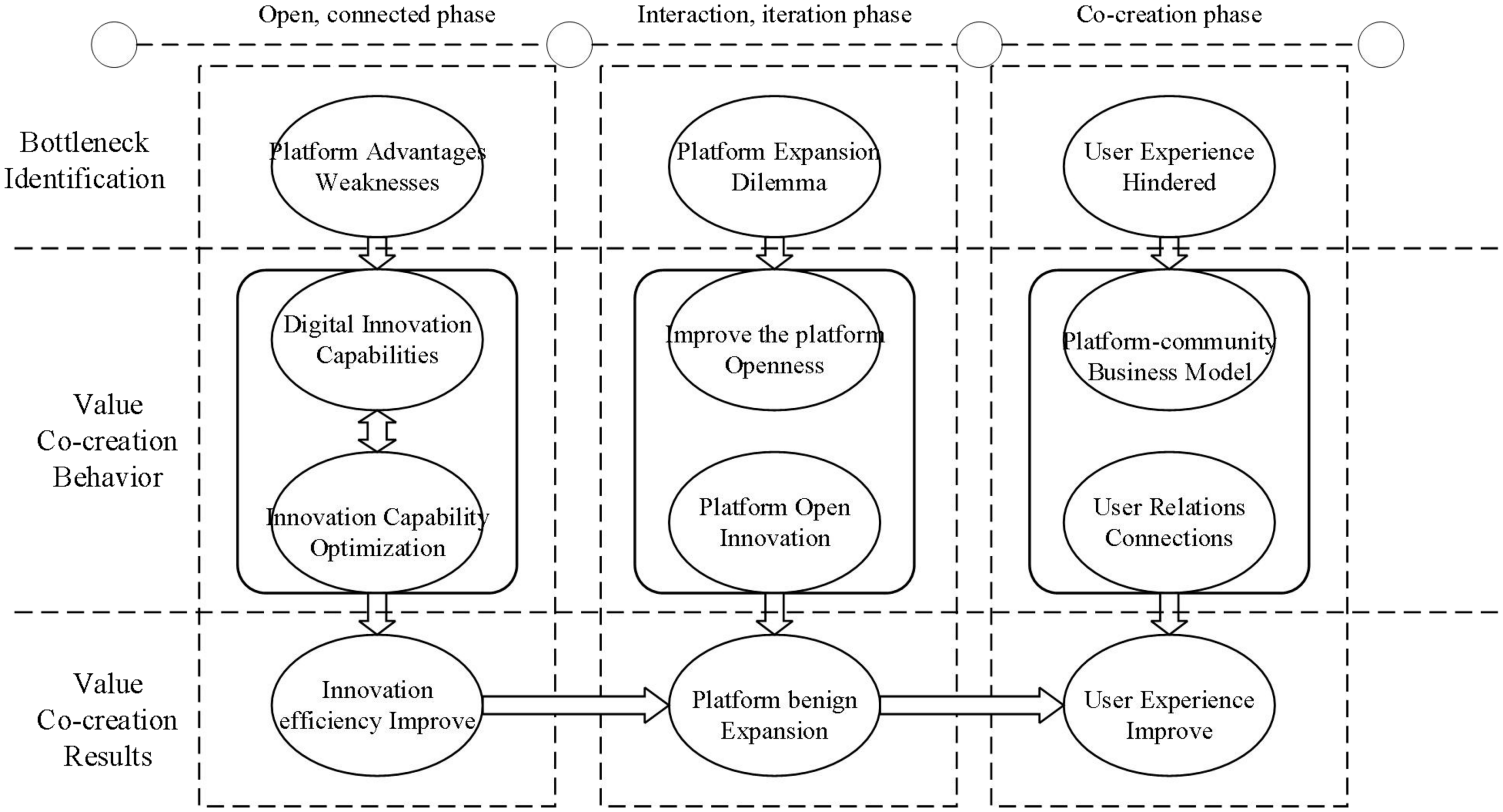
Model of co-creation mechanism of platform companies in the digital innovation ecosystem.

This paper adopts an exploratory case study approach, based on the development of key events in the growth process of the HOPE platform, to explore the intrinsic mechanism of active and passive events on the co-creation of platform enterprises in the digital innovation ecology, and to reveal the important role of digital innovation capability, platform openness, and platform business model innovation in the value co-creation process of platform enterprises (Figure 5).

The mechanism of value co-creation of platform enterprises is reflected in the screening of platform enterprises based on bottleneck problems, the platform implementing different behaviors, and promoting the co-creation of multilateral users on the platform and industrial links. In the open connection stage, the platform solves the problem of weakened platform advantages by continuously improving the digital innovation capability, and ultimately improves the efficiency of enterprise innovation; in the interaction and iteration stage, the platform innovation capability is lacking, so it needs to continuously open the platform and carry out open innovation to break through the platform expansion dilemma and achieve benign platform expansion. At the stage of co-creation, user experience is hindered, and the platform adopts a platform-community business model to connect user relationships and realize user experience improvement.

## Conclusion and outlook

### Research conclusion

Digital innovation capabilities play an important role in the operation of platform companies. Platform enterprises are founded on insufficient platform advantages and a lack of innovation capabilities to assist them to explore and develop. With the continuous application and development of digital technology, platform enterprises have a stronger ability to identify, judge, and control the environment and objective conditions. So they can achieve efficiency improvement and create new business models. At the same time, the development of the market environment requires the platform to use the Internet, big data, and other digital technologies to continuously open itself. And they promote multiparty cooperation between subjects with the advantage of the platform, from “single win” to “win-win.” Enterprises can use the exploratory digital innovation capability to cope with the passive type of events with high intensity and wide spatial diffusion, find innovation opportunities, integrate innovation resources, improve the efficiency of resource sharing, optimize innovation capability, and then provide accurate services to users in a more agile manner. With the continuous development of platform advantages, enterprises also start to expand their development and open their platforms continuously. The more the number of platform participants, the richer the types and the wider the coverage area, a closely connected network will be formed, which helps platform enterprises realize value growth. With the integration of ecological platform information, the information sharing and value co-creation of different data platforms are realized on the open collaborative innovation platform, oriented to the needs of users in dynamic scenarios. The information sharing and value co-creation of different data platforms are realized on the open collaborative innovation platform, oriented to the needs of users in dynamic scenarios. This makes the intelligent dialog between different data possible and further reconstructs the business model of platform enterprises. The limited resources, the inertia of the R&D process, and the delay of market feedback usually lead to a time lag between R&D and market demand. However, by interacting with the community, enterprises can disperse the fragmented and individual demand for information and creative inspiration in the community so that the community can complement the existing resources of enterprises and help enterprises stimulate incremental innovation. They also can break the organizational inertia of enterprises and quickly discover, meet, and even create market demand. The platform-community business model brings the relationship between the platform and users closer, and platform enterprises optimize service quality around users’ needs to improve users’ experience. Platform enterprises can also capture external demands in real-time and take timely countermeasures to promote the platform to create new values.

As an important player in the digital innovation ecosystem, platform enterprises not only provide infrastructure for members in the ecosystem; but also improve their digital innovation capabilities, which can promote interaction among digital subjects, enhance system effectiveness, promote information sharing, and enhance intra-and intrasubject cooperation and system innovation. Platform enterprises enter into multiple business scenarios by empowering participants, and participants make themselves sub-platforms in niche areas with the help of the platform. In this way, they expand their management boundaries, nesting both to expand outward and relying on the network effect of co-creation and a win-win situation. At the same time, it accelerates the platform enterprises to become the dominant organizational form in the innovation ecosystem. The innovative integration and orchestration capabilities of platform companies can reduce transaction costs with suppliers and attract more complementary companies. It can also expand the boundaries of the platform ecosystem, increase the variety of products and services in the ecosystem, and thus attract more users. Platform enterprises promote the development of interaction among internal business entities. The more open the platform is, the more platform enterprises will continue to expand their development, become more flexible in the ecosystem, and gradually realize the digital multilateral platform. Platform enterprises and multilateral subjects together create a highly open and inclusive innovation ecosystem, thus helping the system to enhance its knowledge innovation capability. Digital-driven platform business model innovation can realize mass customization. Through data empowerment, enterprises drive the intrinsic linkage at the level of the entire platform ecosystem, forming a synergy with resource reconfiguration and the release of network effects to design more accurate products for user needs. By the way, enterprises can improve user satisfaction and increase the success rate of technology transformation. Based on the community ecology, the platform enterprises and the community jointly build a platform-community business model with different value propositions, value creation, and value transmission processes. The value proposition of the platform-community business model is generally win-win and empowerment; the value creation is the different forms of innovation of participating subjects; the value transfer is the business value delivered by the community to the platform. The platform-community model promotes more open communication and cooperation among innovation agents, and it speeds up the process of enterprises’ access to external information, resources, and cooperation opportunities. It also enhances the endogenous evolutionary drive of the digital innovation ecosystem.

In the digital innovation ecosystem, the platform enterprise is in the leading position of the platform and at the core of the ecosystem. It leads the construction of the innovation ecosystem, assumes the responsibility of coordinating the relationship of relevant innovation subjects, and formulates system rules and systems. It also effectively identifies platform users’ needs and gathers and integrates internal and external innovation service resources. It also provides users with precise service solutions through collaborative services to ensure the continuity of innovation activities. During system operation, Platform enterprises interact with data resources through the platform to achieve cost reduction and efficiency improvement in business operations. It can strengthen the willingness of enterprises to innovate and promote the sustainable and rapid development of the system. On the other hand, data is the new production factor of the platform economy. The massive data resources gathered by large platforms can quickly open up the upstream and downstream of the industry. They can transform and form a new digital innovation ecosystem. The digital innovation ecosystem enables the data resources on the platform to be mined, reorganized, and integrated. With digital innovation, the data resources on the platform can be mined, reorganized, and integrated, prompting enterprises to participate in the construction of the industry chain ecosystem and thus realize value co-creation. Enterprises connect discrete manufacturers, suppliers, retailers, customers, and other market participants online to improve operational efficiency, while identifying effective demand online to deeply meet user needs and improve user experience. Through their internal construction and external cooperation, the platform enterprises in the digital innovation ecosystem gradually form a self-organized and self-looping value co-creation, forming a data-driven co-creation model. They give full play to the modular nature of the digital innovation ecosystem to help improve the effectiveness of system innovation.

### Shortcomings and prospects

This paper also has certain research limitations, and more scholars are still needed to do further exploration in future research: (1) The digital innovation ecosystem is a complex system of multiple interactions, and with the changes in the social and corporate environment. There are often multiple tensions between platform enterprises and stakeholders, but the existing research lacks systematic analysis. Future research can further explore platform enterprises from the perspective of stakeholders’ Legitimacy construction and building the governance mechanism of platform enterprises. (2) The interactive behaviors in the innovation ecosystem led by platform firms are influenced by the traits and resource characteristics of the digital innovation ecosystem. There are openness and complementarity differences as well as industrial attribute differences in innovation ecosystems led by different platform firms. Future research can open up the mechanism of empowerment differentiation among different types of platform firms. (3) This paper is based on a single-case study approach, and although attention is paid to the typicality and polarization of cases, there are inherent limitations of individual cases. Future research can carry out dynamic case tracking for startups and state-owned enterprises and other industries to compare the impact mechanisms of digital innovation capabilities, platform openness, and platform business model innovation on value co-creation of platform enterprises in different industries.

### Recommendations

The platform economy has changed the market structure and competitive behavior of different industries, and the platform enterprises, as an important factor among them, their platform architecture determines the business model of future enterprises and their product architecture. Therefore, platform enterprises should optimize the design of platform architecture, and accelerate the application of core technologies such as big data, cloud computing, and blockchain in platform enterprises. They also should support and encourage platforms to strengthen innovation and inject vitality into the sustainable development of the ecology.

As the main reliance of the digital innovation ecosystem. On the one hand, platform enterprises should strengthen communication with complementary enterprises in the system. Enterprises need to organize the exchange and communication of system members to promote the smooth flow of knowledge and information. In addition, enterprises should coordinate the optimization of resource allocation and enrich the business system from multiple perspectives and levels, so as to enhance the flexibility of the digital innovation ecosystem and its ability to resist market risks. On the other hand, in order to strengthen the stability of the digital innovation ecosystem, relevant government departments should actively cultivate leading enterprises with strong driving force that can maintain the stable development of the digital innovation ecosystem. At the same time, they should strengthen the cultivation of the core competitiveness of the leading platform enterprises to ensure the sustainable and healthy development of the entire digital innovation ecosystem network.

## Data availability statement

The original contributions presented in the study are included in the article/supplementary material, further inquiries can be directed to the corresponding author/s.

## Author contributions

KF: methodology, writing—original draft, formal analysis. YL and JZ: data analysis, visualization. XG: conceptualization, writing—review and editing, project administration, supervision. ZX and CL: revised and edited the manuscript. All authors have read and agreed to the published version of the manuscript.

## Funding

This research was supported by the National Social Science Foundation of China (19BJY099) and the Shanghai Philosophy and Social Science Planning Project (2020BJB015).

## Conflict of interest

The authors declare that the research was conducted in the absence of any commercial or financial relationships that could be construed as a potential conflict of interest.

## Publisher’s note

All claims expressed in this article are solely those of the authors and do not necessarily represent those of their affiliated organizations, or those of the publisher, the editors and the reviewers. Any product that may be evaluated in this article, or claim that may be made by its manufacturer, is not guaranteed or endorsed by the publisher.
